# Circulating cathelicidin levels correlate with mucosal disease activity in ulcerative colitis, risk of intestinal stricture in Crohn’s disease, and clinical prognosis in inflammatory bowel disease

**DOI:** 10.1186/s12876-017-0619-4

**Published:** 2017-05-12

**Authors:** Diana Hoang-Ngoc Tran, Jiani Wang, Christina Ha, Wendy Ho, S. Anjani Mattai, Angelos Oikonomopoulos, Guy Weiss, Precious Lacey, Michelle Cheng, Christine Shieh, Caroline C. Mussatto, Samantha Ho, Daniel Hommes, Hon Wai Koon

**Affiliations:** 1 Vatche and Tamar Manoukian Division of Digestive Diseases, David Geffen School of Medicine at the University of California Los Angeles, Los Angeles, CA 90095 USA; 2 Department of Medicine, David Geffen School of Medicine at the University of California Los Angeles, Los Angeles, CA 90095 USA; 3 Department of Gastroenterology, First Affiliated Hospital, China Medical University, Shenyang City, Liaoning Province China; 4 Center for Inflammatory Bowel Diseases, Vatche and Tamar Manoukian Division of Digestive Diseases, Department of Medicine, David Geffen School of Medicine, 43-152 Center for Health Sciences Building, 10833 Le Conte Avenue, Los Angeles, CA 90095 USA

**Keywords:** Biomarkers, Complications of IBD, Serologic testing, Prognosis, Mucosal disease activity

## Abstract

**Background:**

Cathelicidin (LL-37) is an antimicrobial peptide known to be associated with various autoimmune diseases. We attempt to determine if cathelicidin can accurately reflect IBD disease activity. We hypothesize that serum cathelicidin correlates with mucosal disease activity, stricture, and clinical prognosis of IBD patients.

**Methods:**

Serum samples were collected from two separate cohorts of patients at the University of California, Los Angeles. Cohort 1 consisted of 50 control, 23 UC, and 28 CD patients. Cohort 2 consisted of 20 control, 57 UC, and 67 CD patients. LL-37 levels were determined by ELISA. Data from both cohorts were combined for calculation of accuracies in indicating mucosal disease activity, relative risks of stricture, and odds ratios of predicting disease development.

**Results:**

Serum cathelicidin levels were inversely correlated with Partial Mayo Scores of UC patients and Harvey-Bradshaw Indices of CD patients. Among IBD patients with moderate or severe initial disease activity, the patients with high initial LL-37 levels had significantly better recovery than the patients with low initial LL-37 levels after 6–18 months, suggesting that high LL-37 levels correlate with good prognosis. Co-evaluation of LL-37 and CRP levels was more accurate than CRP alone or LL-37 alone in the correlation with Mayo Endoscopic Score of UC patients. Low LL-37 levels indicated a significantly elevated risk of intestinal stricture in CD patients.

**Conclusion:**

Co-evaluation of LL-37 and CRP can indicate mucosal disease activity in UC patients. LL-37 can predict future clinical activity in IBD patients and indicate risk of intestinal stricture in CD patients.

**Electronic supplementary material:**

The online version of this article (doi:10.1186/s12876-017-0619-4) contains supplementary material, which is available to authorized users.

## Background

Inflammatory bowel diseases (IBD) such as ulcerative colitis (UC) and Crohn’s disease (CD) are complex immune-mediated disorders associated with heterogeneous disease presentation. Diagnosis and evaluation of disease activity involves imaging and procedures such as ileocolonoscopy, magnetic resonance imaging (MRI), and computed tomography (CT) scans that may be invasive, risky, and costly [1–3]. Thus, these procedures are not performed frequently, and biomarkers are more commonly used for disease activity evaluation. Approximately 10–25% of CD patients develop at least one intestinal stricture, demonstrating the need for an accurate biomarker for detection of stricture [4]. Current IBD biomarkers include C-reactive protein (CRP), erythrocyte sedimentation rate (ESR), and the more IBD-specific fecal calprotectin (FC), which has a greater correlation to inflammatory colitis activity [5]. However, these biomarkers do not consistently demonstrate accuracy in correlation to certain IBD parameters such as mucosal disease activity and strictures [6]. For this reason, accurate IBD biomarkers for IBD disease activity are being sought after actively.

Antimicrobial peptides such as lactoferrin have demonstrated clinical utility as IBD biomarkers [7]. An additional antimicrobial peptide, cathelicidin, is reported to have anti-inflammatory and anti-fibrogenic effects [8–11]. Cathelicidin (also known as hCAP18) is a 18 kDa peptide consisting of 37 amino acids [12]. Its antimicrobial functions depend on permeabilization of bacterial cell membrane with multiple cationic cathelicidin molecules [13]. Cathelicidin is expressed broadly in various tissues such as colonic mucosa [14], breast [15], salivary glands [16], and various kinds of immune cells [17]. Human cathelicidin promoter consists of a vitamin D response element (VDRE) [18]. Oral vitamin D administration has been demonstrated to induce skin cathelicidin expression in patients with atopic dermatitis [19]. Another report demonstrated the association between vitamin D, activation of cathelicidin expression, and tuberculosis infection [20]. Besides antimicrobial functions, cathelicidin possesses anti-inflammatory effect against endotoxin lipopolysaccharide [21]. Other antimicrobial peptides such as hepcidin [22] and beta-defensin-2 [23] do not correlate with IBD disease activity.

Focusing on IBD, Schauber et al. show that colonic cathelicidin (*CAMP*) mRNA expression is increased in ulcerative colitis patients [24]. Animal studies also demonstrated that mice with a cathelicidin deficiency are more susceptible to DSS-mediated colitis than wild-type mice [25]. Low serum cathelicidin levels are associated with sepsis in patients of intensive care units [26] and mortality in cases of severe renal disease [27], while high serum cathelicidin levels are observed in patients with autoimmune diseases such as psoriasis [28] and vasculitis [29]. Collectively, this evidence suggests a general correlation of circulating cathelicidin with infection, inflammation, and autoimmune diseases.

IBD is regarded as an autoimmune disease. It is possible that circulating LL-37 levels are altered in the patients of IBD. Levels of circulating cathelicidin in IBD patients have never been analyzed in literature, and the correlation between cathelicidin and IBD disease activity has not yet been determined. We hypothesize that circulating cathelicidin levels have the potential to accurately indicate IBD disease activity. In this study, we examined serum cathelicidin levels for correlation to IBD disease activity. We compared diagnostic accuracies of LL-37 alone, CRP alone, and combined LL-37 + CRP for indicating various clinical and mucosal disease activity parameters. We evaluated the prognostic capability of serum LL-37 levels in indicating the disease development 6–18 months later and determined the relative risk of intestinal stricture in CD patients with low serum cathelicidin levels.

## Methods

### Patients and samples

Blood was collected from control, UC, and CD patients through procedures established by UCLA. For cohort 1, IBD patients were recruited from a UCLA Gastroenterology clinic, and control samples were obtained from a UCLA Internal Medicine clinic. Serum samples from cohort 1 were prepared by UCLA Department of Pathology. Serum samples from cohort 2 were obtained from UCLA Center for Inflammatory Bowel Diseases Biobank. All blood samples were collected between 2012 and 2015. The blood samples were collected following indicated diagnostic procedures ordered by the physicians. Serum volume availability must be 100 μL or above. Specimen must be in good quality with no sign of hemolysis. For IBD patients, clinical data of follow-up visits at 6–18 months after the initial blood draw were collected for the analysis. The blood samples of cohort 1 and 2 do not overlap.

### Inclusion and exclusion criteria

Inclusion criteria: Samples were obtained from male and female subjects (age 21–67 years). IBD-specific inclusion criteria: The IBD patients sought diagnosis and/or treatment for IBD. IBD diagnosis (UC and CD) was confirmed by board-certified gastroenterologists. Exclusion criteria: No pregnant women, prisoners, or minors under age 18 were included. Patients with concurrent acute infection (CMV, *C. difficile*, *and tuberculosis*) and malignant conditions were not included. IBD patients without follow-up visits at 6–18 months after the initial blood draw were not included. Dr. Koon has already included all qualified blood samples available at the time of ELISA experiments in 2015.

### Assessment of disease activity and presence of stricture

The Partial Mayo Score (PMS) and Harvey Bradshaw Index (HBI) were used to assess the clinical disease activity of UC and CD patients respectively [30, 31]. Evaluation of PMS and HBI was performed on the same day of blood sample collection for LL-37 ELISA, CRP, and other tests. The diagnosis of stricture was determined by imaging procedures or colonoscopy occurring within 1 month of blood sample collection. Mayo Endoscopic Subscore (MES) was used to assess the mucosal disease activity of the UC patients (range 0–3) [32]. All data were collected prospectively.

### LL-37 and C-reactive protein (CRP)

Measurement of LL-37 was performed using an ELISA kit (#HK321, Fisher Scientific, Pittsburgh, PA) at Dr. Koon’s laboratory according to manufacturer’s instructions as previously described [25]. CRP levels were determined by the UCLA Department of Pathology and their values were available at the CareConnect database.

### Statistical analysis

Power analysis has shown that this study required at least 40 patients per group of control, UC, and CD patients to achieve a statistically significant difference of serum LL-37 levels between control (37 ng/ml), UC (56 ng/ml), and CD (58 ng/ml) patients with standard deviation = 21, alpha = 0.5, and power = 0.8. The combined data from two cohorts yielded 70 control, 80 UC, and 95 CD patients total that satisfied this requirement.

LL-37 protein concentration and CRP levels were arranged in order from low to high. Low LL-37 levels indicated moderate and severe disease, and high LL-37 levels indicated remission. High CRP levels indicated moderate and severe disease, and low CRP levels indicated remission. We determined the performance of multiple cut-off points of LL-37 and CRP levels at each disease parameter. The optimized universal cut-off points were shown in this study.

Prevalence of disease, sensitivity, specificity, positive predictive value (PPV), and negative predictive values (NPV), with their 95% confidence intervals (CI) were calculated using a clinical calculator website: http://vassarstats.net/clin1.html. The accuracy of a test was determined by Area under Curve (AUC) of Receiver Operating Characteristics (ROC) using: http://www.rad.jhmi.edu/jeng/javarad/roc/JROCFITi.html. Odds ratio and relative risk were calculated using a clinical calculator website: https://www.medcalc.org/calc/. Results were expressed as mean +/− SEM. Bar graphs and scatter plots were made using Microsoft Excel and GraphPad Prism (San Diego, CA). Unpaired Student’s *t*-tests were used for intergroup comparisons of continuous data and Fisher’s exact tests were used for intergroup comparisons of category data using GraphPad Prism. Significant *p* values are shown in each figure.

## Results

Baseline characteristics are shown in Table 1. Cohort 1 consisted of 50 control patients, 23 UC, and 28 CD patients. Cohort 2 consisted of 20 control patients, 57 UC, and 67 CD patients. Demographic profiles, disease conditions, and medication uses of these two cohorts are comparable, but not the same. Statistical analysis was performed after combining both cohorts. Detailed definitions of Montreal classification of inflammatory bowel disease for general disease phenotypes have been described in a previous report [33].

**Table 1:** Baseline characteristics of cohorts 1 and 2

Baseline Characteristics	Cohort 1			Cohort 2		
	Control	UC	CD	Control	UC	CD
LL-37 Range (ng/mL)	23–79	12–245	13–94	26–70	21–167	13–282
Age at Collection (mean ± SEM)	46 ± 2	40 ± 2	35 ± 2	43 ± 3	37 ± 1	38 ± 2
Age at diagnosis (mean ± SEM)		35 ± 2	30 ± 2		28 ± 1	25 ± 1
Duration of Disease in Years (mean ± SEM)		6 ± 1	6 ± 1		8 ± 1	13 ± 2
Gender (% Male)	42%	35%	25%	40%	56%	52%
Percentage of patients who used biologics		13%	38%		16%	45%
Percentage of patients who used steroids		17%	52%		32%	30%
Percentage of patients who used immunomodulators		9%	14%		21%	38%
Percentage of patients who used 5-aminosalicyclic acid (5-ASA)		74%	24%		58%	24%
Percentage of Current Smoker (%)	20%	13%	21%	20%	7%	12%
C-reactive protein (CRP) Levels (mg/L) (mean ± SEM)		1.18 ± 0.42	4.77 ± 1.43		0.75 ± 0.16	1.59 ± 0.39
UC Partial Mayo Score (mean ± SEM)		2.09 ± 0.47			3.04 ± 0.29	
UC Mayo Endoscopic Score (mean ± SEM)		1.64 ± 0.19			1.13 ± 0.14	
UC Partial Mayo Score 6–18 months later (mean ± SEM)		2.40 ± 0.52			1.59 ± 0.28	
Montreal Classification of UC:						
UC ulcerative proctitis E1 (%)		38%			16%	
UC left sided colitis E2 (%)		24%			59%	
UC pancolitis E3 (%)		29%			22%	
CD Harvey Bradshaw Index (mean ± SEM)			3.67 ± 0.77			4.22 ± 0.44
CD Harvey Bradshaw Index 6–18 months later (mean ± SEM)			2.54 ± 0.65			1.38 ± 0.26
Montreal Classification of CD:						
CD Age at diagnosis less than 16 years A1 %			12%			27%
CD Age at diagnosis 17–40 years A2 %			59%			60%
CD Age at diagnosis over 40 years A3 %			29%			12%
CD ileal L1 (%)			56%			23%
CD colonic L2 (%)			6%			15%
CD ilealcolonic L3 (%)			38%			63%
CD isolated upper digestive tract L4 (%)			13%			7%
CD non-stricturing, non-penetrating B1 (%)			43%			57%
CD stricturing B2 (%)			50%			24%
CD penetrating B3 (%)			11%			27%
CD perianal disease P (%)			11%			0%
	50	23	28	20	57	67

### Serum cathelicidin negatively correlates with clinical disease activity of UC patients

**Fig. 1:**
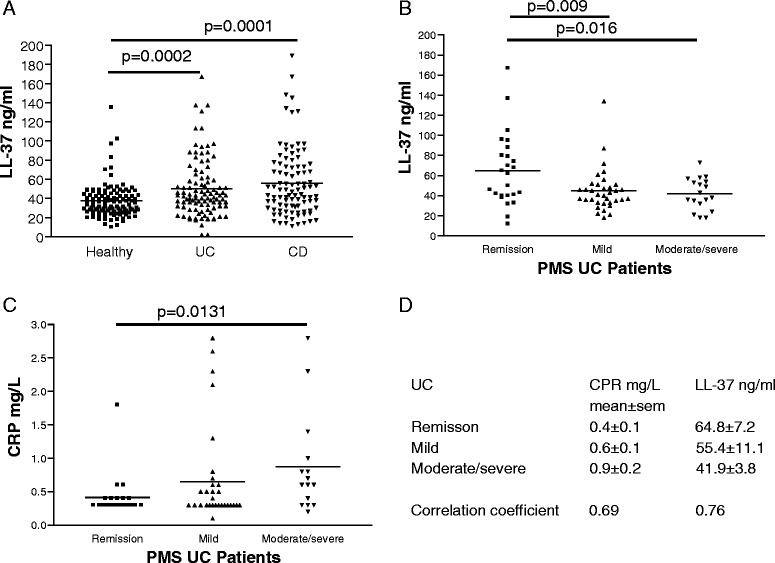
Serum LL-37 levels negatively correlate with Partial Mayo Score of UC patients. **a** Serum LL-37 levels of normal, UC, and CD patients. **b** Scatter plot shows the negative correlation of PMS values and serum LL-37 levels of UC patients. **c** Scatter plot shows the positive correlation of PMS values and serum CRP levels of UC patients. **d** Mean and SEM values of CRP and LL-37 in UC patients. Correlation coefficients of CRP/LL-37 versus PMS of UC patients

Our results showed UC and CD patients had significantly increased serum cathelicidin levels with 56 ± 5 ng/ml and 58 ± 4 ng/ml (mean ± sem) respectively, compared to control patients averaging 37 ± 2 ng/ml (Fig. 1a). Cathelicidin levels were not associated with age at collection, age at diagnosis, duration of diseases, gender, or smoking habits in both UC and CD patients (data not shown). Serum cathelicidin levels were inversely proportional to PMS of UC patients, consistent with its known anti-inflammatory effect (Fig. 1b & d). As expected, serum CRP levels were directly proportional to PMS of UC patients (Fig. 1c-d).

**Fig. 2:**
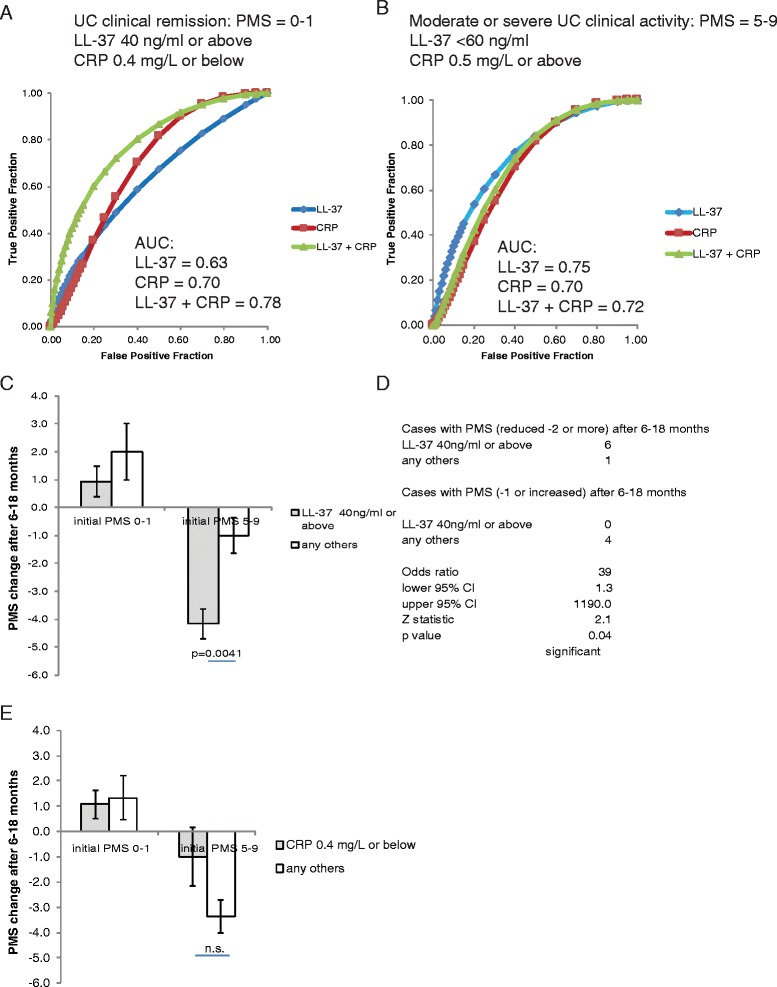
Serum LL-37 levels alone can predict UC clinical prognosis. **a** ROC curves with AUC values show the accuracy of using LL-37 alone, CRP alone, and both for indicating UC clinical remission. **b** ROC curves with AUC values demonstrate the accuracy of using LL-37 alone, CRP alone, and both for indicating moderate or severe UC. **c** A bar graph shows the PMS changes of the UC patients at 6–18 months after the initial blood draw and LL-37 determination. **d** The odds ratio of PMS changes among UC patients with PMS 5–9 initially. (E) A bar graph shows the PMS changes of the UC patients at 6–18 months after the initial blood draw and CRP determination

We used two optimized universal cut-off points of LL-37 and CRP levels for indicating the clinical disease activity in UC patients from two cohorts. A combination of high LL-37 levels and low CRP levels was slightly more accurate than LL-37 alone or CRP alone in indicating UC clinical remission (Fig. 2a). Sensitivity and specificity of the LL-37 + CRP combined test in indicating UC clinical remission are 0.71 and 0.80 respectively (Additional file 1: Figure S1A). LL-37 alone was slightly more accurate than CRP alone in indicating moderate and severe UC clinical disease activity (Fig. 2b). Sensitivity and specificity of the LL-37 + CRP combined test in indicating moderate and severe UC clinical disease activity are 0.69 and 0.78 respectively (Additional file 1: Figure S1B).

### Circulating LL-37 levels are better than CRP levels in indicating future clinical activity of UC patients

To evaluate the future disease development of UC patients, we analyzed PMS values that were assessed by the physicians between 6 and 18 months after the initial blood draw for LL-37 and CRP measurement. Since some of the patients were initially in clinical remission while others began with moderate or severe disease activity, we evaluated these two groups of patients separately. The result shows that all patients in clinical remission initially (PMS = 0–1) remained in remission or slightly worsened to mild disease activity after 6–18 months (Fig. 2c). For the patients with moderate or severe disease initially, the high LL-37 group had significantly greater reduction in PMS values than the low LL-37 group (4.2 ± 0.5 versus 1.0 ± 0.6), suggesting improved future clinical activity (Fig. 2c). These patients with high LL-37 levels were significantly more likely to have a PMS reduction of 2 points or more in approximately 1 year, with an odds ratio equal to 39 (Fig. 2d). We found no association between LL-37 levels and use of medication from the day of blood draw (0 month) throughout monitoring period (6–18 months, average =12 months) (Additional file 2: Figure S2A). Among the steroid-free UC patients, the high LL-37 group still had significantly greater reduction of PMS values than the low LL-37 group (4.0 ± 0.7 versus 1.5 ± 0.5) (Additional file 2: Figure S2B). Both findings suggested that the improved future clinical activity of the high LL-37 group was not influenced by use of any specific group of medication.

On the other hand, there was no statistically significant difference in PMS reduction after 6–18 months between high and low CRP groups (Fig. 2e). Our study suggests that LL-37 is more accurate than CRP in indicating future clinical activity of moderate or severe UC patients.

### Co-evaluation of LL-37 and CRP levels indicate UC mucosal disease activity accurately

**Fig. 3:**
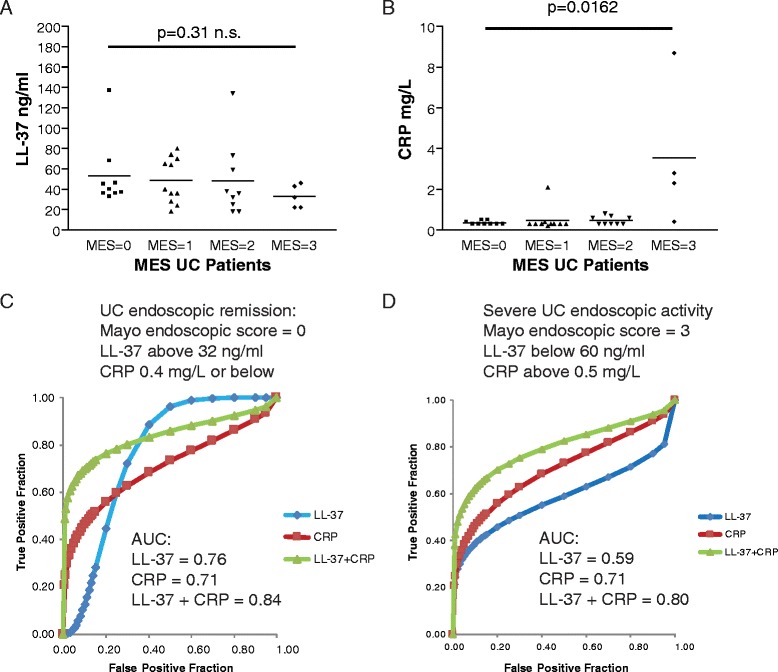
Pairing LL-37 levels with CRP levels can indicate UC mucosal disease activity with greater accuracy than using either alone. **a** Scatter plot shows the negative correlation of Mayo endoscopic scores and serum LL-37 levels of UC patients. **b** Scatter plot shows the positive correlation of Mayo endoscopic scores and serum CRP levels of UC patients. **c** ROC curves with AUC values demonstrate the accuracy of using LL-37 alone, CRP alone, and both for indicating UC endoscopic remission. **d** ROC curves with AUC values demonstrate the accuracy of using LL-37 alone, CRP alone, and both for indicating severe UC endoscopic disease activity

LL-37 levels were inversely proportional to Mayo endoscopic scores (Fig. 3a). Although the difference of serum LL-37 levels between MES = 0 and MES = 3 was not statistically significant, we observed that all UC patients with severe mucosal disease activity (MES = 3) had LL-37 levels below 60 ng/ml while all UC patients with endoscopic remission (MES = 0) had LL-37 levels above 32 ng/ml (Fig. 3a). Therefore, we set up two different universal LL-37 cut-off points for indicating the mucosal disease activity in UC patients from two cohorts. CRP levels were directly proportional to Mayo endoscopic score (Fig. 3b). The difference of serum CRP levels between patients with MES = 0 and patients with MES = 3 was statistically significant (Fig. 3b).

In the evaluation of UC endoscopic remission, a combination of high LL-37 (above 32 ng/ml) levels and low CRP (0.4 mg/L or below) levels (AUC = 0.84) were more accurate than LL-37 alone (AUC = 0.76) or CRP alone (AUC = 0.71) (Fig. 3c). The LL-37 + CRP combined test (0.38) and LL-37 alone (0.38) had slightly better specificities than CRP alone (0.30) (Additional file 3: Figure S3A).

A combination of low LL-37 (below 60 ng/ml) levels and high CRP (above 0.5 mg/L) levels (AUC = 0.80) were more accurate than LL-37 alone (AUC = 0.59) or CRP alone (AUC = 0.71) in indicating severe endoscopic disease activity in UC patients (Fig. 3d). The LL-37 + CRP combined test (0.85) had better specificity than LL-37 alone (0.32) and CRP alone (0.77) (Additional file 3: Figure S3B). Co-evaluation of the LL-37 test and the CRP test can provide an accurate assessment of mucosal disease activity, especially the indication of severe UC endoscopic activity. We found no association between LL-37 levels, UC disease location (E1-3), and use of medication (Additional file 3: Figure S3C).

### Serum cathelicidin negatively correlates with clinical disease activity of CD patients

**Fig. 4:**
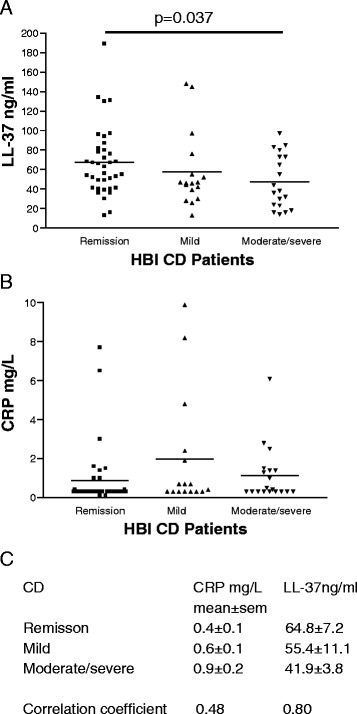
Serum LL-37 levels negatively correlate with Harvey Bradshaw Indices of CD patients. **a** Scatter plot shows the negative correlation between HBI values and serum LL-37 levels of CD patients. **b** Scatter plot shows no correlation of HBI values and serum CRP levels of CD patients. **c** Mean and SEM values of CRP and LL-37 in CD patients. Correlation coefficients of CRP/LL-37 versus HBI of CD patients

**Fig. 5:**
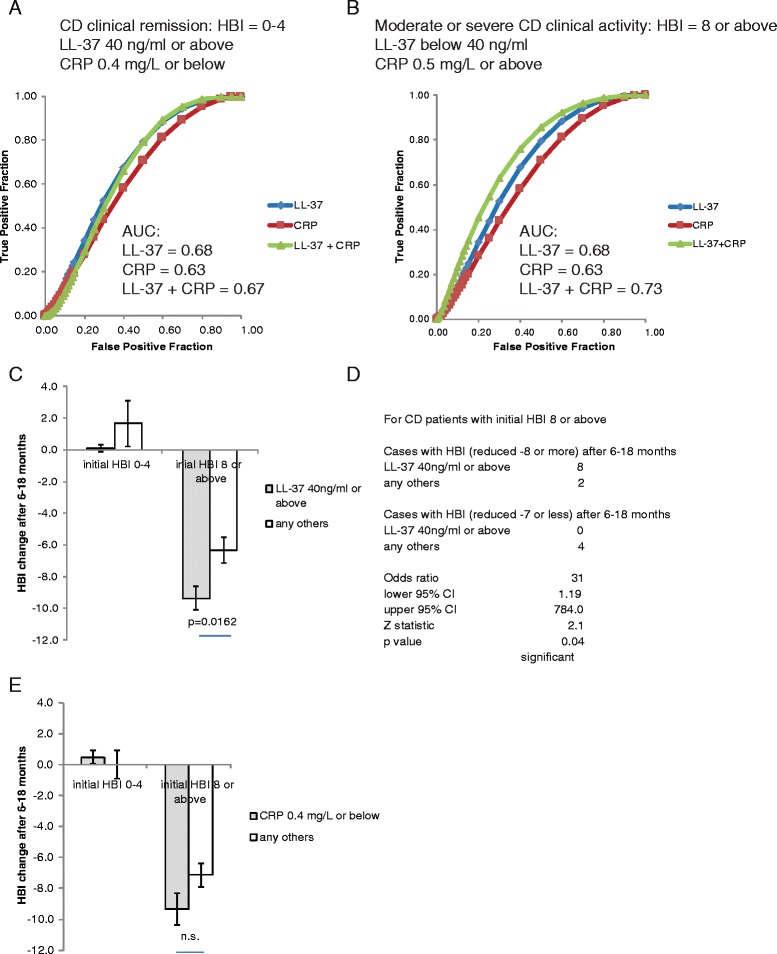
Serum LL-37 levels alone can predict CD clinical prognosis. **a** ROC curves with AUC values show the accuracy of using LL-37 alone, CRP alone, and both for indicating CD clinical remission. **b** ROC curves with AUC values demonstrate the accuracy of using LL-37 alone, CRP alone, and both for indicating moderate or severe CD. **c** A bar graph shows the HBI changes of the CD patients at 6–18 months after the initial blood draw and LL-37 determination. **d** The odds ratio of HBI changes among CD patients with initial HBI 8 or above. **e** A *bar graph* shows the HBI changes of the CD patients at 6–18 months after the initial blood draw and CRP determination

Serum cathelicidin levels were inversely proportional to HBI values of CD patients (Fig. 4a & c). There was no correlation between HBI values and CRP levels (Fig. 4b & c). We used a universal LL-37 cut-off point to differentiate levels of clinical disease activity in CD patients from both cohorts. For both indication of CD clinical remission and moderate/severe CD clinical disease activity, LL-37 alone was equally as accurate as CRP alone (Fig. 5a-b). Co-evaluation of both parameters did not further increase accuracy. The prevalence, sensitivity, specificity, PPV, NPV, and AUC data were shown in Additional file 4: Figure S4A-B. We found no association between LL-37 levels, age (A1-3), disease location (L1-4), disease behavior (B1-3/P), or use of medication at the time of blood draw (Additional file 4: Figure S4C). The mucosal disease activity of CD patients was assessed by simple endoscopic score (SES-CD); however, there was no statistically significant correlation between LL-37 and mucosal disease activity in CD patients (the high LL-37 group 3.11 ± 0.4 vs. the low LL-37 group 4.14 ± 1.6, mean ± sem).

### Circulating LL-37 levels are better than CRP levels in indicating future clinical activity of CD patients

To assess future disease development of CD patients, we analyzed their HBI values collected between 6 and 18 months after the initial blood draw for LL-37 and CRP measurement. We evaluated the patients with initial clinical remission and moderate/severe disease separately. The result shows that the patients initially in remission (HBI = 0–4) remained in remission after 6–18 months, regardless of LL-37 levels (Fig. 5c). For the patients with moderate or severe disease initially, the high LL-37 group had significantly reduced HBI values (−9.4 ± 0.7) at the second measurement compared to the low LL-37 group (−6.3 ± 0.8), suggesting better future clinical activity (Fig. 5c). These patients with high LL-37 levels were significantly likely to have HBI reduction of 8 points or more in 6–18 months, with an odds ratio equal to 31 (Fig. 5d). Most of these high LL-37 patients reached clinical remission. On the other hand, the same group of CD patients with CRP levels 0.4 mg/L or below tended to have similar future clinical activity as those with CRP above 0.4 mg/L, and the difference between groups was not statistically significant (Fig. 5e). LL-37 was better than CRP in indicating future clinical activity of moderate or severe CD patients. There was no association between initial disease activity, LL-37 levels, and use of medication from the day of blood draw (0 month) throughout monitoring period (6–18 months, average =12 months) (Additional file 5: Figure S5A). Among the steroid-free CD patients, the high LL-37 group still had significantly greater reduction of HBI values than the low LL-37 group (8.8 ± 0.48 versus 6.3 ± 0.80) (Additional file 5: Figure S5B). The improved future clinical activity of the CD patients in the high LL-37 group did not appear to be influenced by use of any specific group of medication.

### Low serum LL-37 levels but not high CRP levels indicate an elevated risk of stricture

Among CD patients, the low LL-37 group had significantly higher relative risk (RR = 1.8) than the high LL-37 group in developing intestinal stricture (Table 2a). Meanwhile, the relative risk of developing stricture in high CRP groups was not elevated in comparison to low CRP groups (Table 2b). LL-37 was superior to CRP in reflecting the relative risk of intestinal stricture in CD patients. The details of prevalence, sensitivity, specificity, PPV, and NPV data of the LL-37 test (below 40 ng/ml) are shown in Table 2c.

**Table 2:** Low serum LL-37 levels indicate an elevated relative risk of the presence of intestinal strictures in CD patients.

A		B	
LL-37 below 40 ng/ml		CRP 0.5 mg/ml or above	
cases w ith stricture	13	cases w ith stricture	16
cases w ithout stricture	15	cases w ithout stricture	25
LL-37 40 ng/ml or above		CRP 0.4 mg/L or below	
cases w ith stricture	17	cases w ith stricture	14
cases w ithout stricture	48	cases w ithout stricture	38
Relative risk	1.7752	Relative risk	1.4495
low er 95% CI	1.0	low er 95% CI	0.8
upper 95% CI	3.1	upper 95% CI	2.6
p-value	0.0485	p-value	0.2167
	significant		not significant
C			
test positive	LL-37 below 40 ng/ml		
test negative	any others		
	mean	(95% CI interval)	
prevalence	0.32	0.23	0.43
sensitivity	0.43	0.26	0.62
specificity	0.76	0.64	0.86
PPV	0.46	0.28	0.66
NPV	0.74	0.61	0.84

(A) The relative risk of the low LL-37 group with the presence of stricture among CD patients. (B) The relative risk of high CRP group with the presence of stricture among CD patients. (C) Prevalence, sensitivity, specificity, PPV, and NPV values of LL-37 test in indicating the presence of stricture

## Discussion

This study determined the optimal LL-37 cut-off points for indicating clinical disease activity, mucosal disease activity, stricture, and future clinical activity. The LL-37 test should only be used to indicate disease activity after the IBD diagnosis is confirmed because IBD patients with moderate and severe disease activity may also have serum cathelicidin levels as low as control patients. In animal models, systemic overexpression of cathelicidin suppresses colitis while systemic deficiency of endogenous cathelicidin worsens colitis [11, 25]. This explains why the cathelicidin level is inversely proportional to disease activity in IBD.

Colonic epithelial cells express cathelicidin, and colonic cathelicidin mRNA expression is shown to be increased in UC patients but not CD patients [24]. However, the contribution of colonic cathelicidin expression to circulating cathelicidin levels and overall IBD disease activity is not known, and it is beyond the scope of this study. Our previous animal study demonstrated that cathelicidin genotype of bone-marrow derived cells plays a significant role in dextran sulfate-mediated colitis [25]. This evidence suggests that immune cells may modulate colitis development via cathelicidin expression.

Although physicians can assess clinical disease activity of patients by asking questions about IBD-related symptoms, they often rely on one or more biomarkers (CRP, ESR, and FC) to provide an additional reference in the assessment of overall disease activity and drug responses. As shown in Figs. 2 and 5, LL-37 and CRP have similar accuracy in indicating clinical disease activity. The LL-37 ELISA experiment is inexpensive, quick to produce results, and easy to perform. Collection of blood samples for LL-37 tests may be more convenient than collection of fecal samples for calprotectin tests as patients may not be able to provide stool samples at the time of clinical visit. The LL-37 test may be a promising alternative to the CRP test.

Co-evaluation of LL-37 and CRP showed 18% improvement of accuracy in indicating UC endoscopic remission and 13% improvement in indicating moderate or severe UC endoscopic disease activity when compared to CRP alone (Fig. 3). It is common to assess mucosal disease activity using biomarkers since ileocolonoscopy is not frequently performed. As CRP testing is not highly accurate in the evaluation of mucosal disease activity, this combined test optimizes the existing CRP test. The LL-37 + CRP test may be useful when invasive ileocolonoscopy is not advisable. This LL-37 + CRP combined test (AUC = 0.8) may be more accurate than FC (AUC = 0.639) in indicating endoscopic remission (MES = 0), according to a recent study [34]. Unfortunately, we were not able to compare the performance of LL-37 + CRP with FC side-by-side since the FC tests were not frequently performed in our cohorts. Similar to a previous study [35], we found no correlation between HBI and CRP levels in CD patients.

Another strength of this LL-37 test is the prediction of future clinical activity. The LL-37 test is superior to the CRP test in predicting recovery within 6–18 months (Figs. 2 and 5). For IBD patients with moderate or severe clinical disease activity at the time of blood draw, patients with high LL-37 levels showed better recovery than patients with low LL-37 levels after 6–18 months. There was no association between LL-37 levels and use of medications in both cohorts (data not shown). We believe that the good recovery in high LL-37 group is independent of response to any particular kind of medication. Co-evaluation of LL-37 and CRP does not improve the predictive correlation (data not shown). The potential capability of LL-37 in predicting UC endoscopic disease activity prognosis is promising. We found a trend of good recovery from mucosal disease in high LL-37 UC group (data not shown); however, the data are insufficient for a full analysis at this moment.

There is no well-established biomarker for stricture diagnosis. The LL-37 test (low LL-37 group) can indicate an elevated risk of the presence of stricture with high specificity (Table 2a-c). Although the LL-37 test is not intended to diagnose the presence of stricture due to low sensitivity, our finding presents a novel method to help physicians identify high-risk CD patients for further screening. Based on the anti-fibrogenic effect of cathelicidin in animal models [11], it is reasonable to expect that low circulating cathelicidin expression correlates with an elevated risk of stricture. CD stricture can be classified into inflammatory stricture or non-inflammatory fibrostenotic stricture. However, there is no clear consensus in the approach of differentiation between these two types [36]. Unfortunately, there was no distinction between stricture types made by the physicians for the patients included in this study. We aim to reevaluate the potential use of LL-37 levels in indicating the inflammatory stricture versus fibrostenotic stricture in the future once adequate guideline have been established. In addition, LL-37 was not associated with the relative risk of fistula occurrence in CD patients at the time of blood draw (data not shown). This study already partially explored the association of LL-37 levels and stricture development. It may be interesting to determine whether LL-37 levels can predict development of other intestinal complications in the future.

This report provides novel evidence for the value of circulating cathelicidin levels as a new IBD biomarker. Circulating cathelicidin levels inversely correlate with clinical and mucosal disease activity in UC and CD patients. High LL-37 levels alone predict good clinical prognosis. Co-existence of a low LL-37 level and clinical remission indicates an elevated risk of intestinal stricture. Besides the diagnostic utility of cathelicidin in IBD patients, cathelicidin has been demonstrated to have potential as a novel therapeutic strategy against colitis [10], intestinal fibrosis [11], and colitis-associated colon cancer [37]. Systemic administration of cathelicidin peptide to patients is unsafe due to its hemolytic property [38]. For this reason, we are exploring a new therapeutic strategy involving oral administration of a non-peptide cathelicidin mimic for treating colitis and its complications.

## Conclusions

In conclusion, the serum cathelicidin test exhibits accuracy in indicating IBD disease activity. Cathelicidin levels may be used to optimize the performance of existing CRP tests, and can serve as an indicator of stricture and future clinical activity for IBD patients.

## Additional files

Additional file 1: **Figure S1.** Data analysis of UC clinical activity. Prevalence, sensitivity, specificity, PPV, NPV, and AUC values of ROC curves of LL-37 test alone, CRP test alone, and both in indicating (A) UC clinical remission and (B) moderate or severe UC clinical activity. (PDF 15 kb)

Additional file 2: **Figure S2.** Use of medication of UC patients. (A) A table showing the use of medication on the day of blood draw (0 month) and the average end-point of monitoring period (12 months). (B) A bar graph shows the changes in PMS of the UC patients at 6–18 months after the initial blood draw and LL-37 determination. These UC patients did not use steroids throughout the 6–18 month monitoring period. (PDF 79 kb)

Additional file 3: **Figure S3.** Data analysis of UC endoscopic activity. Prevalence, sensitivity, specificity, PPV, NPV, and AUC values of ROC curves of LL-37 test alone, CRP test alone, and both in indicating (A) endoscopic remission and (B) severe UC endoscopic activity. (C) There was no association between LL-37 levels, disease location, and use of medication at the time of blood draw among UC patients. The Montreal classification of UC is shown. (PDF 81 kb)

Additional file 4: **Figure S4.** Data analysis of CD clinical activity. Prevalence, sensitivity, specificity, PPV, NPV, and AUC values of ROC curves of LL-37 test alone, CRP test alone, and both in indicating (A) CD clinical remission and (B) moderate or severe CD clinical activity. (C) There was no association between LL-37 levels, age, disease location, disease behavior, and use of medication at the time of blood draw among UC patients. The Montreal classification of CD is shown. (PDF 40 kb)

Additional file 5: **Figure S5.** Use of medication of CD patients. (A) A table showing the use of medication on the day of blood draw (0 month) and the average end-point of monitoring period (12 months). (B) A bar graph shows the changes in HBI of the CD patients at 6–18 months after the initial blood draw and LL-37 determination. These CD patients did not use steroid medication throughout the 6–18 month monitoring period. (PDF 80 kb)

## Abbreviations

AUC: Area under curve
CD: Crohn’s disease
CRP: C-reactive protein
ESR: Erythrocyte sedimentation rate
FC: Fecal calprotectin
HBI: Harvey Bradshaw Index
IBD: Inflammatory bowel disease
LL-37: Human cathelicidin
NPV: Negative predictive value
PMS: Partial Mayo score
PPV: Positive predictive value
ROC: Receiver operating characteristics
UC: Ulcerative colitis
