# Supplementary material for: Circulating cathelicidin levels correlate with mucosal disease activity in ulcerative colitis, risk of intestinal stricture in Crohn’s disease, and clinical prognosis in inflammatory bowel disease
Source: BMC Gastroenterol. 2017 May 12;17:63. doi: 10.1186/s12876-017-0619-4 (PMC5427565; doi:10.1186/s12876-017-0619-4)
Supplement: Supplementary file 1 — Figure S1. Data analysis of UC clinical activity. Prevalence, sensitivity, specificity, PPV, NPV, and AUC values of ROC curves of LL-37 test alone, CRP test alone, and both in indicating (A) UC clinical remission and (B) moderate or severe UC clinical activity. (PDF 15 kb) [file 12876_2017_619_MOESM1_ESM.pdf]

Supplementary Figure 1

A

UC clincial remission: PMS = 0-1

	LL-37 40ng/ml or above			CRP 0.4mg/L or below			LL-37 40ng/ml or above + CRP 0.4mg/L or below		
test positive	LL-37 40ng/ml or above			CRP 0.4mg/L or below			LL-37 40ng/ml or above + CRP 0.4mg/L or below		
test negative	any others			any others			any others		
	mean	(95% CI interval)		mean	(95% CI interval)		mean	(95% CI interval)	
prevalence	0.35	0.25	0.47	0.32	0.22	0.44	0.32	0.22	0.44
sensitivity	0.81	0.60	0.93	0.83	0.62	0.95	0.71	0.49	0.87
specificity	0.50	0.35	0.65	0.48	0.34	0.62	0.80	0.66	0.89
PPV	0.47	0.32	0.62	0.43	0.29	0.59	0.63	0.42	0.80
NPV	0.83	0.64	0.93	0.86	0.66	0.95	0.85	0.71	0.93
AUC	0.63			0.70			0.78		

B

Moderate or severe UC clinical activity: PMS = 5-9

test positive	LL-37 <60ng/ml			CRP 0.5mg/L or above			LL-37 <60ng/ml + CRP 0.5mg/L or above		
test negative	any others			any others			any others		
	mean	(95% CI interval)		mean	(95% CI interval)		mean	(95% CI interval)	
prevalence	0.22	0.13	0.33	0.18	0.10	0.29	0.22	0.13	0.33
sensitivity	0.94	0.68	1.00	0.62	0.32	0.85	0.69	0.41	0.88
specificity	0.31	0.20	0.45	0.67	0.54	0.78	0.78	0.64	0.87
PPV	0.27	0.17	0.41	0.29	0.14	0.49	0.46	0.26	0.67
NPV	0.95	0.72	1.00	0.89	0.76	0.96	0.90	0.78	0.96
AUC	0.75			0.70			0.72		
