# Supplementary material for: Circulating cathelicidin levels correlate with mucosal disease activity in ulcerative colitis, risk of intestinal stricture in Crohn’s disease, and clinical prognosis in inflammatory bowel disease
Source: BMC Gastroenterol. 2017 May 12;17:63. doi: 10.1186/s12876-017-0619-4 (PMC5427565; doi:10.1186/s12876-017-0619-4)

Supplementary Figure 2

A

Percentage of medication uses (%)		biologics 0mo	biologics 6-18mo	steroids 0mo	steroids 6-18mo	immunomodulators 0mo	immunomodulators 6-18mo	5-ASA 0mo	5-ASA 6-18mo
initial PMS=0-1	LL-37 40ng/ml or above	14	15	7	8	29	30	86	100
initial PMS=0-1	any others	33	33	33	33	0	0	67	33
initial PMS=5-9	LL-37 40ng/ml or above	0	33	50	0	17	50	67	33
initial PMS=5-9	any others	0	20	0	20	20	20	60	80

B

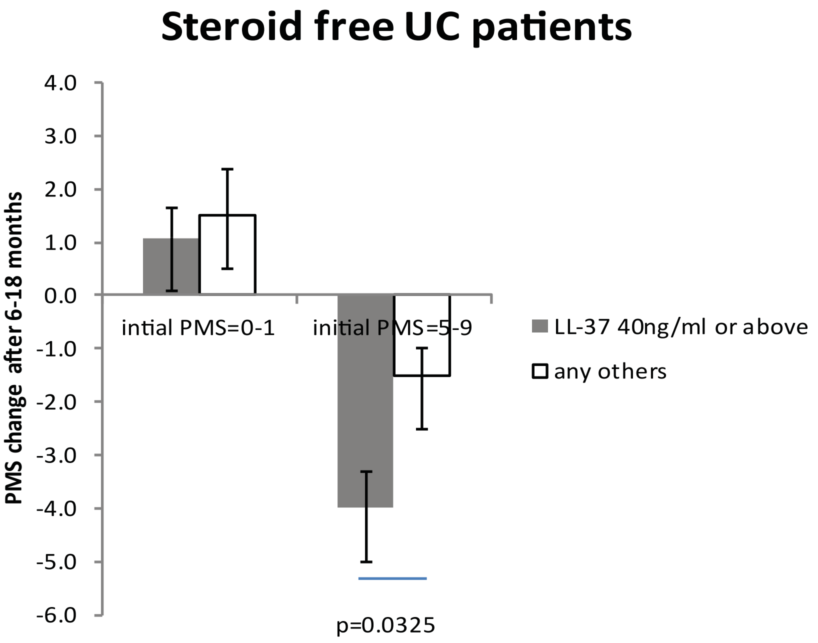

Supplement: Supplementary file 2 — Figure S2. Use of medication of UC patients. (A) A table showing the use of medication on the day of blood draw (0 month) and the average end-point of monitoring period (12 months). (B) A bar graph shows the changes in PMS of the UC patients at 6–18 months after the initial blood draw and LL-37 determination. These UC patients did not use steroids throughout the 6–18 month monitoring period. (PDF 79 kb) [file 12876_2017_619_MOESM2_ESM.pdf]
