# Supplementary material for: Circulating cathelicidin levels correlate with mucosal disease activity in ulcerative colitis, risk of intestinal stricture in Crohn’s disease, and clinical prognosis in inflammatory bowel disease
Source: BMC Gastroenterol. 2017 May 12;17:63. doi: 10.1186/s12876-017-0619-4 (PMC5427565; doi:10.1186/s12876-017-0619-4)
Supplement: Supplementary file 3 — Figure S3. Data analysis of UC endoscopic activity. Prevalence, sensitivity, specificity, PPV, NPV, and AUC values of ROC curves of LL-37 test alone, CRP test alone, and both in indicating (A) endoscopic remission and (B) severe UC endoscopic activity. (C) There was no association between LL-37 levels, disease location, and use of medication at the time of blood draw among UC patients. The Montreal classification of UC is shown. (PDF 81 kb) [file 12876_2017_619_MOESM3_ESM.pdf]

Supplementary Figure 3

A

UC endoscopic remission: Mayo endoscopic score = 0

test positive	LL-37 above 32ng/ml	CRP 0.4mg/L or below	LL-37 above 32ng/ml + CRP 0.4mg/L or below
test negative	any others	any others	any others

	LL-37 mean	(95% CI interval)		CRP mean	(95% CI interval)		LL-37 + CRP mean	(95% CI interval)	
prevalence	0.27	0.14	0.46	0.23	0.11	0.43	0.30	0.15	0.50
sensitivity	1.00	0.63	1.00	0.71	0.30	0.95	1.00	0.63	1.00
specificity	0.38	0.20	0.59	0.30	0.14	0.53	0.38	0.19	0.61
PPV	0.38	0.20	0.59	0.24	0.09	0.48	0.41	0.21	0.63
NPV	1.00	0.63	1.00	0.78	0.40	0.96	1.00	0.60	1.00
AUC	0.76			0.71			0.84		

B

Severe UC endoscopic activity: Mayo endoscopic score = 3

test positive	LL-37 below 60ng/ml	CRP above 0.5 mg/L	LL-37 below 60ng/ml + CRP above 0.5 mg/L
test negative	any others	any others	any others

	LL-37 mean	(95% CI interval)		CRP mean	(95% CI interval)		LL-37 + CRP mean	(95% CI interval)	
prevalence	0.15	0.06	0.33	0.13	0.04	0.32	0.13	0.04	0.32
sensitivity	1.00	0.46	1.00	0.75	0.22	0.99	0.75	0.22	0.99
specificity	0.32	0.17	0.52	0.77	0.56	0.90	0.85	0.64	0.95
PPV	0.21	0.08	0.43	0.33	0.09	0.69	0.43	0.12	0.80
NPV	1.00	0.63	1.00	0.95	0.74	0.99	0.96	0.76	1.00
AUC	0.59			0.71			0.80		

C

		**E1**	**E2**	**E3**	**biologics**	**steroids**	**immunomodulators**	**5-ASA**
LL-37 32 ng/ml or below	count	0	5	1	2	2	2	3
	percentage	0	83	17	33	33	33	50
LL-37 above 32 ng/ml	count	8	26	12	5	10	9	26
	percentage	19	60	28	12	23	21	60

		**E1**	**E2**	**E3**	**biologics**	**steroids**	**immunomodulators**	**5-ASA**
LL-37 below 60ng/ml	count	7	24	7	7	16	7	23
	percentage (%)	18	60	18	16	37	16	54
LL-37 60ng/ml or above	count	1	7	6	2	2	5	10
	percentage (%)	7	50	43	14	14	36	71

General disease phenotypes: Montreal classification of UC

E1	Ulcerative proctitis
E2	Left side UC (distal UC)
E3	Extensive UC (pancolitis)
