# Supplementary material for: Circulating cathelicidin levels correlate with mucosal disease activity in ulcerative colitis, risk of intestinal stricture in Crohn’s disease, and clinical prognosis in inflammatory bowel disease
Source: BMC Gastroenterol. 2017 May 12;17:63. doi: 10.1186/s12876-017-0619-4 (PMC5427565; doi:10.1186/s12876-017-0619-4)
Supplement: Supplementary file 4 — Figure S4. Data analysis of CD clinical activity. Prevalence, sensitivity, specificity, PPV, NPV, and AUC values of ROC curves of LL-37 test alone, CRP test alone, and both in indicating (A) CD clinical remission and (B) moderate or severe CD clinical activity. (C) There was no association between LL-37 levels, age, disease location, disease behavior, and use of medication at the time of blood draw among UC patients. The Montreal classification of CD is shown. (PDF 40 kb) [file 12876_2017_619_MOESM4_ESM.pdf]

Supplementary Figure 4

A CD clinical remission: HBI = 0-4

test positive	LL-37 40ng/ml or above			CRP 0.4mg/L or below			LL-37 40ng/ml or above + CRP 0.4mg/L or below		
test negative	any others			any others			any others		
	mean	(95% CI interval)		mean	(95% CI interval)		mean	(95% CI interval)	
prevalence	0.52	0.40	0.64	0.52	0.40	0.64	0.52	0.40	0.64
sensitivity	0.84	0.68	0.93	0.74	0.57	0.86	0.63	0.46	0.78
specificity	0.43	0.27	0.60	0.49	0.32	0.66	0.69	0.51	0.83
PPV	0.62	0.47	0.74	0.61	0.45	0.75	0.69	0.51	0.83
NPV	0.71	0.48	0.88	0.63	0.42	0.80	0.63	0.46	0.78
AUC	0.68			0.63			0.67		

B Moderate or severe CD clinical activity: HBI = 8 or above

test positive	LL-37 below 40ng/ml			CRP 0.5mg/L or above			LL-37 below 40ng/ml + CRP 0.5mg/L or above		
test negative	any others			any others			any others		
	mean	(95% CI interval)		mean	(95% CI interval)		mean	(95% CI interval)	
prevalence	0.25	0.16	0.36	0.26	0.17	0.38	0.25	0.16	0.36
sensitivity	0.56	0.31	0.78	0.42	0.21	0.66	0.33	0.14	0.59
specificity	0.80	0.67	0.89	0.65	0.51	0.77	0.95	0.84	0.99
PPV	0.48	0.26	0.70	0.30	0.14	0.50	0.67	0.31	0.91
NPV	0.85	0.71	0.93	0.76	0.61	0.87	0.81	0.69	0.90
AUC	0.68			0.63			0.73		

C

		A1	A2	A3	L1	L2	L3	L4	B1	B2	B3	P	biologics	steroids	immunomodulators	5-ASA
LL-37 below 40ng/ml	count	5	10	3	5	1	10	1	8	6	7	0	7	1	8	2
	percentage (%)	28	56	17	28	6	56	6	44	33	24	0	39	6	44	11
LL-37 40ng/ml or above	count	13	31	5	9	11	29	3	30	10	11	0	24	19	18	14
	percentage (%)	27	64	10	18	22	59	6	61	20	22	0	49	39	37	29

General disease phenotypes: Montreal classification of CD	
A1	CD Age at diagnosis less than 16 years
A2	CD Age at diagnosis 17-40 years
A3	CD Age at diagnosis over 40 years
L1	CD ileal
L2	CD colonic
L3	CD ilealcolonic
L4	CD isolated upper digestive tract
B1	CD non-stricturing, non-penetrating
B2	CD stricturing
B3	CD penetrating
P	CD perianal disease
