# Supplementary material for: Circulating cathelicidin levels correlate with mucosal disease activity in ulcerative colitis, risk of intestinal stricture in Crohn’s disease, and clinical prognosis in inflammatory bowel disease
Source: BMC Gastroenterol. 2017 May 12;17:63. doi: 10.1186/s12876-017-0619-4 (PMC5427565; doi:10.1186/s12876-017-0619-4)

Supplementary Figure 5

A

Percentage of medication uses (%)		biologics 0mo	biologics 6-18mo	steroids 0mo	steroids 6-18mo	immunomodulators 0mo	immunomodulators 6-18mo	5-ASA 0mo	5-ASA 6-18mo
initial HBI=0-4	LL-37 40ng/ml or above	38	52	48	0	38	48	38	24
initial HBI=0-4	any others	60	80	20	40	20	20	0	40
initial HBI≥8	LL-37 40ng/ml or above	78	63	44	0	22	25	11	13
initial HBI≥8	any others	33	67	0	0	50	50	0	0

B

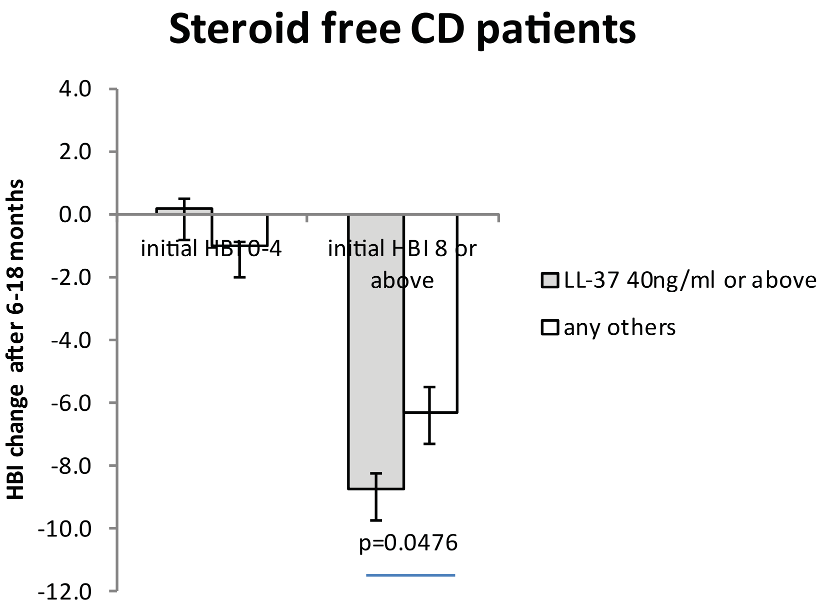

Supplement: Supplementary file 5 — Figure S5. Use of medication of CD patients. (A) A table showing the use of medication on the day of blood draw (0 month) and the average end-point of monitoring period (12 months). (B) A bar graph shows the changes in HBI of the CD patients at 6–18 months after the initial blood draw and LL-37 determination. These CD patients did not use steroid medication throughout the 6–18 month monitoring period. (PDF 80 kb) [file 12876_2017_619_MOESM5_ESM.pdf]
